# “You Sleep, You Die”: A Rare Clinical Case of Ondine's Curse after Posterior Fossa Surgery

**DOI:** 10.1155/2023/3113428

**Published:** 2023-09-11

**Authors:** Mitesh Karn, Basant Kumar Mahato, Ranjan Sah, Dipendra Kandel, Shabal Sapkota

**Affiliations:** ^1^Department of Neurosurgery, Gandaki Medical College Teaching Hospital and Research Center, Pokhara, Nepal; ^2^Department of Neurosurgery, Al Tadawi Specialty Hospital, Dubai, UAE

## Abstract

Ondine's curse is a rare condition in which breathing is preserved while awake, but there is absence of autonomic control of ventilation. It is a potentially fatal complication that may result rarely from surgery in posterior fossa in area close to respiratory centers. We describe a patient with posterior fossa hemangioblastoma who underwent subtotal resection via telovelar approach and developed acquired Ondine's curse postoperatively. The patient's presentation and management are described. Besides that, Ondine's is a great example of how modern medicine is linked to ancient literature, and thus, its anecdotal history is also described.

## 1. Introduction

Ondine's curse or central hypoventilation syndrome (CHS) is a rare condition wherein alveolar hypoventilation occurs due to impaired autonomic control of ventilation, and the voluntary control remains intact. These patients “forget to breathe” while asleep. Two types of CHS are classically described: congenital and acquired. Congenital CHS is the commoner type and is caused by a variant in PHOX2B gene [1]. Acquired CHS is related to trauma, tumor, infection, infarction, surgery, and other conditions usually involving the brainstem [2]. Though rare, Ondine's curse can occur after neurosurgical procedures performed around sensitive brain areas containing respiratory centers. We, herein, describe a rare case of acquired CHS that occurred after posterior fossa surgery for hemangioblastoma.

## 2. Case Presentation

**Figure 1:**
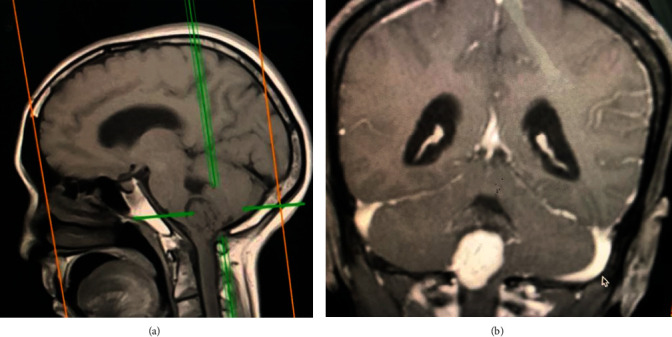
Sagittal T1 (a) and coronal T2 (b) MRI sequences showing a well-defined mass lesion measuring 2.2^∗^2.0 cm at the region of cerebellomedullary cistern.

**Figure 2:**
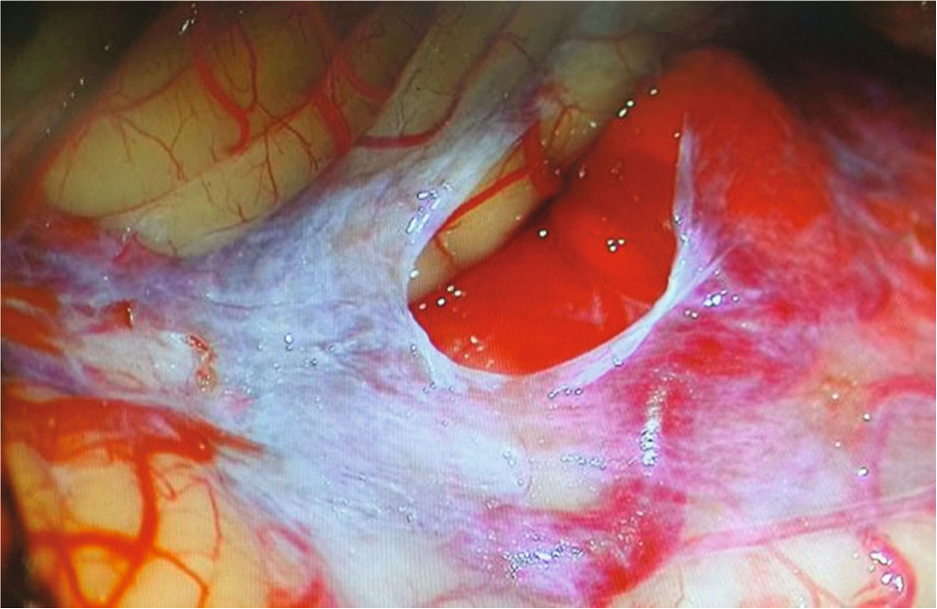
Operative image showing the posterior fossa tumor.

A 56-year-old patient (body mass index, 22.7), a known case of hypothyroidism and hypertension under medication, presented to neurosurgery outpatient clinic with complaints of dysphagia for two months and tingling sensation associated with left sided hemiparesis (Medical Research Council grade 4/5) for two years. Neurological examination revealed positive cerebellar signs and horizontal nystagmus. Bowel and bladder habits were normal, and the patient had a pleasant sleep. Magnetic resonance imaging (MRI) of the brain showed a well-defined mass lesion measuring 2.2^∗^2.0 cm at the cerebellomedullary cistern (Figure 1). The lesion was hypointense in T1WI, hyperintense in T2WI, and fluid-attenuated inversion recovery (FLAIR) sequences with intense postcontrast enhancement. The outlet of 4th ventricle was slightly obstructed by the lesion leading to dilatation of fourth ventricle and temporal horns of lateral ventricle. A posterior fossa craniectomy with subtotal excision of the lesion was carried out via telovelar approach (Figure 2). Histopathology confirmed the lesion to be hemangioblastoma.

### 2.1. Postoperative Course

**Figure 3:**
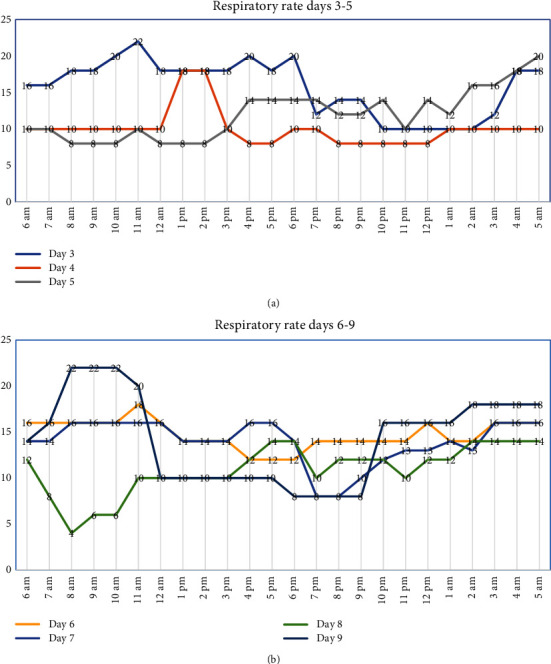
Respiratory pattern during postoperative day three through five (a) and day six through nine (b).

On the first postoperative day, our patient was fully sedated and paralyzed and maintained on volume control/assist control mode. On the second postoperative day, weaning and spontaneous breathing trial were carried out. Our patient was extubated later that day with start of nasogastric feeding, with her respiratory rate being 14-17 breaths per minute. In postoperative day three, sedation was stopped and our patient's respiratory rate ranged from 10 to 22 breaths per minute, rates dipping through the night. Arterial blood gas (ABG) taken while the patient was awake showed a pCO_2_ 30.9 mmHg and pO_2_ 84 mmHg and during sleep showed a pCO_2_ 55 mmHg and pO_2_ 60 mmHg. Close continuous monitoring of the patient's respiration and sleep showed that our patient developed apneic episodes when asleep but was able to breathe when she was made to wake up (Figure 3). There was no change in postoperative neurological finding. Based on clinical evaluation and lab parameters, a diagnosis of acquired CHS was made. Our patient underwent a tracheostomy on the fifth postoperative day. She was started on respiratory stimulants acetazolamide 250 mg and medroxyprogesterone 20 mg, both thrice daily, and aminophylline 400 mg twice daily without any improvement in automatic breathing. After 14 days of hospital stay, she was discharged against medical advice for financial reasons.

## 3. Discussion

The tale of Ondine is among one of the most fascinating myths in the field of medicine. The play was written by Jean Giraudoux in 1938 based on 1811 novel written by German Friedrich de la Motte. Ondine/Undine was a water nymph blessed with eternal youth and immortality, only until she gave birth to a human child. She fell in love with a man, married him, and had a baby. During their marriage, the man swore to her, “My every waking breath shall be my pledge of love and faithfulness to you!” When Ondine grew old, her beauty faded. One day, she caught her husband cheating on her for some younger woman. Aghast, she cursed him, “You pledged faithfulness to me with your every waking breath, and I accepted that pledge; so be it; for as long as you are awake, you shall breathe, but should you ever fall asleep, your breathing shall cease” [2, 3]. In modern medicine, it was in 1962 when Severinghaus and Mitchell first used the eponym Ondine's curse in postcordotomy patients to describe a failure of automaticity of respiratory centers [4]. These patients would breathe normally while awake but required artificial ventilation during sleep. Historically, this term has been applied to several respiratory and neurological disorders such as sleep apnea and hypoventilation syndrome. At present, it is usually applied to congenital CHS [5].

The proposed criteria for diagnosis of Ondine's curse include hypercapnia during non-REM sleep, normal pO_2_, or ABG during period of voluntary breathing, hypoventilation during sleep with normal breathing while awake, and exclusion of other pulmonary diseases which can mimic this condition [6]. These criteria are only proposed once, and there is no absolute agreement on these. The single most important thing for diagnosis is the evidence that the patient cannot breathe automatically but can breathe on command [7]. In our case, there was a clear evidence of hypoventilation during sleep, resolution of breathing while awake, and absence of any cardiopulmonary conditions or drug administration that would mimic the disease leading to diagnosis of acquired Ondine's curse.

The brainstem contains major respiratory centers in pons and medulla with descending connections up to cervical cord region. Any lesion or surgical trauma to these sensitive areas can produce respiratory abnormalities, including acquired Ondine's curse. Neurosurgical procedures around these areas can rarely cause this condition, and it has been reported in a few instances as a complication of posterior fossa surgery [8]. Prompt clinical diagnosis is very important since this condition is invariably fatal without intervention. Our patient might have developed this complication for two reasons. First, the use of surgical patties to delineate the tumor from floor of fourth ventricles might have caused pressure over the respiratory centers. Secondly, we coagulated on a major vessel that was traversing over the tumor that might have caused ischemia.

Treatment is mainly supportive and aimed at maintaining adequate oxygenation and ventilation, both during sleep and wakefulness, and at maximizing quality of life. Though various modalities such as respiratory stimulants, diaphragmatic pacing and negative pressure ventilation are described in the management of Ondine's; mechanical ventilation remains the mainstay of treatment [9]. Most of the patients require intensive care support and positive pressure ventilation either by a tracheostomy or noninvasively via facemask. Very rarely, spontaneous recovery has also been reported.

In conclusion, we can say that Ondine's curse is a great example of how modern medicine is linked to ancient art and literature. Acquired Ondine's curse is a rare but potentially fatal complication of posterior fossa surgery. Vigilant postprocedural monitoring is the key to diagnosis and timely management. Intensive care and respiratory support constitute the basis of treatment. Familiarity of this condition among neurosurgeons, neurointensivists, and anesthesiologists may improve patient outcomes.

## Data Availability

Other clinical data or figures supporting diagnosis or management are available from the authors upon request.

## References

[1] Trang H., Dehan M., Beaufils F., Zaccana I., Amiel J., Gaultier C. (2005). The French congenital central hypoventilation syndrome registry: general data, phenotype, and genotype. *Chest*.

[2] Demartini Z., Maranha Gatto L. A., Koppe G. L., Francisco A. N., Guerios E. E. (2020). Ondine’s curse: myth meets reality. *Sleep Medicine: X*.

[3] Nannapaneni R., Behari S., Todd N. V., Mendelow A. D. (2005). Retracing “Ondine’s curse.”. *Neurosurgery*.

[4] Severinghaus J., Mitchell R. (1962). Ondine’s curse: failure of respiratory center automaticity while awake. *Clinical Research*.

[5] Orrego-González E., Medina-Rincón G. J., Martínez-Gil S., Botero-Meneses J. S. (2020). Ondine’s curse: the origin of the myth. *Arquivos de Neuro-Psiquiatria*.

[6] Pedroso J. L., Baiense R. F., Scalzaretto A. P., Neto P. B., Teixeira De Gois A. F. T., Ferraz M. E. (2009). Ondines curse after brainstem infarction. *Neurology India*.

[7] Farajirad E., Farajirad M., Amini S., Zare R. (2015). Sleep apnea syndrome after posterior fossa surgery: a case of acquired Ondine’s curse. *Iranian Journal of Otorhinolaryngology*.

[8] Matsuyama M., Nakazawa K., Katou M. (2009). Central alveolar hypoventilation syndrome due to surgical resection for bulbar hemangioblastoma. *Internal Medicine*.

[9] Cielo C. M., Marcus C. L. (2014). Central hypoventilation syndromes. *Sleep Medicine Clinics*.

